# Error-Rate Estimation Based on Multi-Signal Flow Graph Model and Accelerated Radiation Tests

**DOI:** 10.1371/journal.pone.0161378

**Published:** 2016-09-01

**Authors:** Wei He, Yueke Wang, Kefei Xing, Wei Deng, Zelong Zhang

**Affiliations:** School of Mechatronics Engineering and Automation, National University of Defense Technology, Changsha, Hu Nan Province, China; Beihang University, CHINA

## Abstract

A method of evaluating the single-event effect soft-error vulnerability of space instruments before launched has been an active research topic in recent years. In this paper, a multi-signal flow graph model is introduced to analyze the fault diagnosis and meantime to failure (MTTF) for space instruments. A model for the system functional error rate (SFER) is proposed. In addition, an experimental method and accelerated radiation testing system for a signal processing platform based on the field programmable gate array (FPGA) is presented. Based on experimental results of different ions (O, Si, Cl, Ti) under the HI-13 Tandem Accelerator, the SFER of the signal processing platform is approximately 10^−3^(error/particle/*cm*^2^), while the MTTF is approximately 110.7 h.

## Introduction

With the wide application of very-large-scale integration(VLSI) on space instruments, along with the decline of device feature size and rise of operation frequency, the single event effect(SEE) is becoming a serious safety problem [1, 2]. It is a hot and difficult task on evaluating the SEE soft error vulnerability of space instruments.

Error rate is the vulnerability parameter which has been modeled and studied by plenty of scientists and engineers. Static and dynamic cross sections are modeled and measured for single device, such as Static Random Access (SRAM), SRAM-based Field Programmable Gate Array (FPGA), and Digital Signal Processor (DSP). A space instrument is comprised of numerous devices. Although cross section of each device can be obtained, it is still difficult to calculate the error rate of the system.

There are four kinds of traditional methods to study the problem, they are onboard testing [3], accelerated radiation testing [4], fault injection [5, 6], and analytical method. Onboard testing is real application testing, the failure and error of space instrument can be observed and recorded when it is in space environment. However, it is costly and time-intensive. Accelerated radiation testing is the primary method for investigating SEE vulnerability of space instrument before launched. The devices are irradiated by heavy ions or protons which are generated by accelerator. It is costly and time-wasted also. Fault injection is a method that the soft error is generated and injected by non-radiation approach. It depends on the system fault mode and fault model. Its accuracy is limited and time-intensive.

Analytical method is developed to evaluate SEE soft error vulnerability of system by modeling and analysis without physical experiment, it becomes a popular method in recent years. A reliability model of SRAM-based FPGA is presented to estimate the design-specific probability of failure, failure rate, and meantime to failure (MTTF) [7, 8]. It is only useful to evaluate a single FPGA circuit.

However, the above methods are not suitable to evaluate SEE vulnerability for space instruments. The reason is that the methods can not model the interlink state of devices, and can not operate at the early design phase. The multi-signal flow graph (MSFG) is adopted to analyze those problems [9, 10]. MSFG model can present multi-fault factors in accordance with a hierarchical classification, which is based on the equipment maintenance level and physical structure.

This paper focuses on the modeling and measurement of error rate of space instruments. The remainder of this paper is organized as follows. In Section II, the modeling method based on multi-signal flow graph is proposed, and SEE vulnerability of a signal processing platform is evaluated. In Section III, the model for obtaining the error rate is proposed. In Section IV, accelerated radiation testing is presented, and the experimental results are discussed. The last part is conclusion.

## Modeling based on Multi-Signal Flow Graph

### Theory of multi-signal flow graph

Multi-signal flow graph is a hierarchical directed graph. Its application includes the testability design for complex systems, and fault diagnosis analysis [11, 12]. A multi-signal flow graph model has the following formal elements:

$$MSFG=\left\{C,S,T,P,E\right\}$$

It includes a finite set of components

$$C=\{c_{1},c_{2},...,c_{L}\}$$

A set of signals

$$S=\{s_{1},s_{2},...,s_{K}\}$$

A finite set of n available tests

$$T=\{t_{1},t_{2},...,t_{N}\}$$

A set of P available test points

$$P=\{P_{1},P_{2},...,P_{R}\}$$

A directed graph

$$DG=\left\{M,P,E\right\}$$

E which represents the set of the system structure directed edge.

In a MSFG model, the dependency between tests and failure sources is computed by capturing relative signals between tests and components. A fault-test dependency matrix can be modeled as diagnostic knowledge. The fault-test dependency matrix is as follows:

$$\begin{array}{c} d_{m\times n}=\left[\begin{array}{cccc} d_{11} & d_{12} & ... & d_{1n}\\ d_{21} & d_{22} & ... & d_{21}\\ ... & ... & ... & ...\\ d_{m1} & d_{m1} & ... & d_{mn} \end{array}\right] \end{array}$$

In the matrix, rows represent tests and columns represent components. If the *c*_*i*_ fault can be detected by test *t*_*j*_, *d*_*ij*_ = 1, otherwise, *d*_*ij*_ = 0.

In order to evaluate the SEE vulnerability for complex electronic systems, particularly those consist of VLSIs, such as SRAM-based FPGA and DSP. The multi-signal flow graph is adopted to research the problem on account of the following reason:

1. Multi-signal flow graph modeling clearly corresponds to an actual physical system. This modeling has advantages in capturing the necessary functional signals without requiring explicit knowledge of system. It is therefore capable of large-scale complex system modeling and the model development is more efficient.
2. Multi-signal flow graph modeling can be applied during the whole design phase. To address increasing application demands and system knowledge, the MSFG can be modeled based on the original structure and knowledge by adding signals to the model.
3. Fault diagnosis and orientation can be achieved by multi-signal flow graph modeling. On one hand, the SEE soft error is generated in the SRAM cell and propagates through device, circuit, and system, which may induce the system function failure. On the other hand, multi-signal flow graph modeling can be used to check the fault mode from upper to lower levels by adding testing points and searching for the SEE vulnerability component.

### Signal processing platform

In this part, a typical signal processing platform which consists of typical VLSIs is introduced.

**Fig 1:**
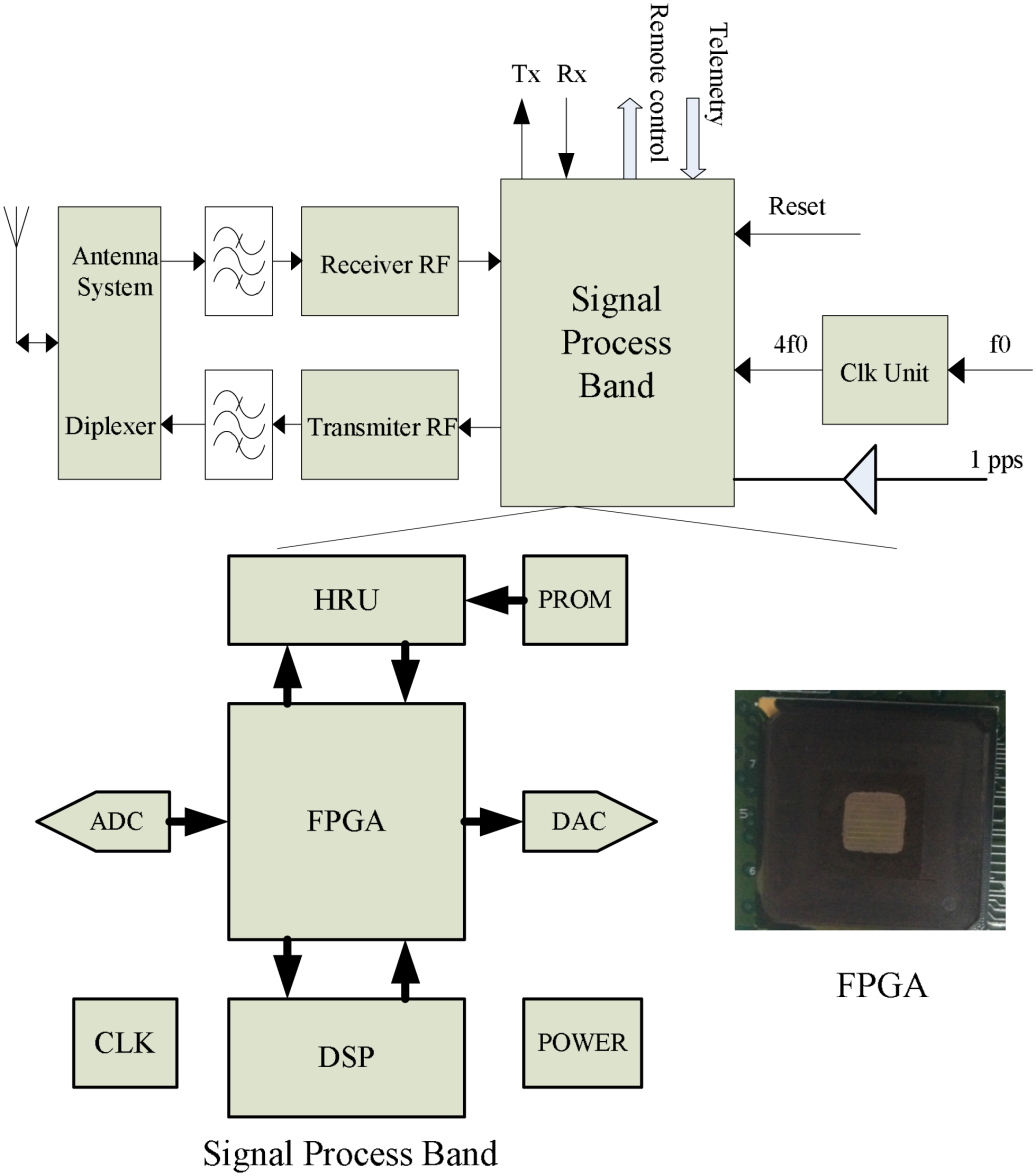
Structure of the transponder.

As shown in Fig 1, the signal processing platform is an important part of the telemetry, tracking, and command (TT&C) satellite system. It is the key platform for linking the ground control station and satellite. The signal processing baseband is the core of the signal processing platform. It adopts the software-defined radio (SDR) concept which is based on the FPGA+DSP structure. The FPGA is a Xilinx Virtex-II XC2V1000-BG575, and DSP is TMS320C6415. A high reliability unit (HRU) is used to manage and monitor the FPGA and DSP. The working schedule is described as following. In FPGA, the uplink digital signal is received after analog-to-digital converter (ADC) sampling, and digital down converter (DDC) is performed, forming two IQ orthogonal signals which are sent to DSP. In DSP, the uplink signal is captured and performs the phase locked loop (PLL), digital locked loop (DLL), and frequency locked loop (FLL) filter calculating. The aim is to evaluate code delay and doppler frequency, so that we can calculate the distance between satellite and ground station. The remote command is demodulated and sent to other satellite equipment. The downlink signal is sent to the digital-to-analog converter (DAC).

### MSFG Modeling

Generally, the MSFG modeling can be done based on the following steps:

1. To get information of the system, includes its structure, the signal flow, and function module, etc.
2. Hierarchical partitioning for the system. Based on the structure and function of the system, a set of components can be obtained.
3. To declare the fault mode for each component. The fault mode is the function failure mode.
4. Adding signals to the components and test points based on the structure and functions of the object system.
5. Building up the multi-signal flow graph model, simulating the model based on TEAMS software, and evaluating the SEE vulnerability.

**Fig 2:**
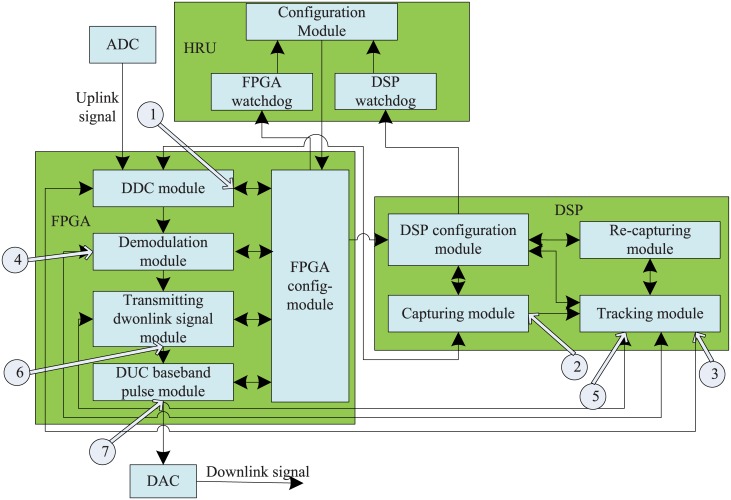
Testing structure of signal processing platform.

The whole multi-signal flow graph of the signal processing platform is shown in Fig 2.

The first hierarchical partition of the system is ADC, HRU, FPGA, DSP, and DAC. The set of components (C1–C12) is the second hierarchical partition of the system. The baseband is divided into a set of components based on the structure and function: C = {C1, C2, C3, C4, C5, C6, C7, C8, C9, C10, C11, C12} = {Configuration module, FPGA watchdog module, DSP watchdog module, capturing module, tracking module, recapturing module, DSP configuration module, DDC module, demodulation module, transmitting downlink signal module, baseband pulse module, and FPGA managing module}.

For the signal processing platform, the multi-signal flow graph model is outlined as follows. The baseband is divided into a set of components based on the structure and function: C = {C1, C2, C3, C4, C5, C6, C7, C8, C9, C10, C11, C12} = {Configuration module, FPGA watchdog module, DSP watchdog module, capturing module, tracking module, recapturing module, DSP configuration module, DDC module, demodulation module, transmitting downlink signal module, baseband pulse module, and FPGA managing module}. The first hierarchical partition of the system is ADC, HRU, FPGA, DSP, and DAC. The set of components (C1–C12) is the second hierarchical partition of the system.

The testing signal set is: S = {S1, S2, S3, S4, S5, S6, S7} = {Normal state, uplink signal cannot be tracked, uplink signal tracked time is overtime, downlink signal blackout, phrase-frequency offset, error code rate, and downlink signal cannot be normally received}. The (S2–S7) is the fault mode for each component. The dependency matrix is shown in Table 1. If the signal can be affected by the component, the value is 1; otherwise, it is 0.

**Table 1:** System component Index of the function.

		C1	C2	C3	C4	C5	C6	C7	C8	C9	C10	C11	C12
S1	The state of system	1	1	1	1	1	0	1	1	1	0	0	1
S2	Uplink signal Tracking	0	0	0	1	1	0	0	1	0	0	0	0
S3	The tracked time of the uplink signal	0	0	0	1	1	0	0	0	0	0	0	0
S4	The downlink signal blackout	0	0	0	0	1	1	0	1	0	1	0	0
S5	The offset of phrase-frequency	0	0	0	0	0	0	0	0	1	1	0	0
S6	The error code rate	0	0	0	0	0	0	0	0	1	0	0	0
S7	The receiving of downlink signal	0	0	0	0	0	0	0	0	0	0	1	0

All targets of the system can be detected by design; thus, the set of tests is T = {t1, t2, t3, t4, t5, t6, t7} = {State of the system, status of whether the uplink signal can be tracked, tracked time of the uplink signal, status of whether the downlink signal is blackout, detecting of the phrase frequency, error code rate, and status of whether the downlink signal can be received}.

There are seven testing points. The set is P = {P1, P2, P3, P4, P5, P6, P7}. The testing point is a setting in the internal position of the device, which implements the signal processing function, especially for SRAM-based FPGA and DSP. The testing points are shown in Fig 2. The dependency matrix is shown in Table 2. In traditional MSFG model, the quantization relationship between two modules is 1 or 0, because it supposes that fault is certain and one fault can lead to another fault. But SEE soft error in complex electronic system is uncertain, fault-coupling. So we introduce fuzzy probability instead of the certain 1 or 0.

**Table 2:** System component Index of the function.

		P1	P2	P3	P4	P5	P6	P7
S1	The state of system	λ_1_	λ_1_	λ_1_	λ_1_	λ_1_	λ_0_	λ_1_
S2	Uplink signal Tracking	λ_0_	λ_0_	λ_1_	λ_0_	λ_1_	λ_0_	λ_0_
S3	The tracked time of the uplink signal	λ_0_	λ_0_	λ_1_	λ_0_	λ_1_	λ_0_	λ_0_
S4	The downlink signal blackout	λ_0_	λ_0_	λ_0_	λ_1_	λ_0_	λ_1_	λ_1_
S5	The offset of phrase-frequency	λ_0_	λ_0_	λ_0_	λ_0_	λ_0_	λ_1_	λ_1_
S6	The error code rate	λ_0_	λ_0_	λ_0_	λ_0_	λ_0_	λ_1_	λ_1_
S7	The receiving of downlink signal	λ_0_	λ_0_	λ_0_	λ_0_	λ_0_	λ_0_	λ_1_

### Simulation based on TEAMS

TEAMS is an X-Window-based software tool that integrates methodology and algorithms in an easy-to-use graphical user interface. TEAMS minimizes the life-cycle cost of a system by aiding the system designer and test engineer in embedding testability features, including built-in test requirements in the system design.

**Fig 3:**
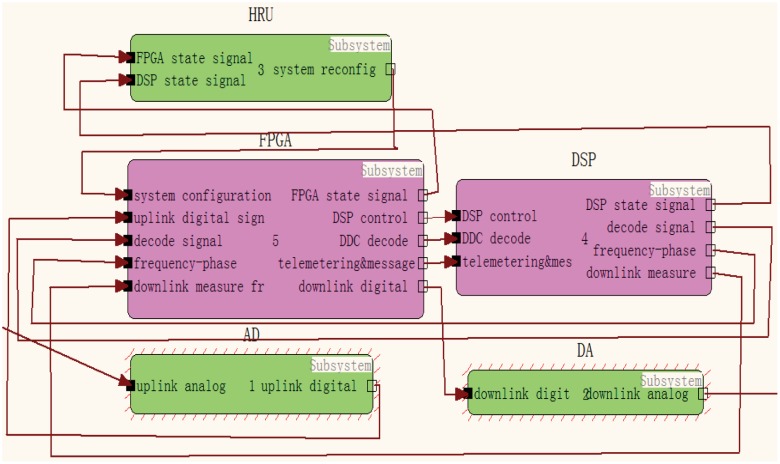
Multi-signal model of platform based on TEAMS.

The MSFG model of the platform using TEAMS is established based on the hierarchical partition, which is shown in Fig 2. The interlink information of different devices (HRU, FPGA and DSP) is shown, also the interlink module information of FPGA and DSP. Fig 3 depicts the top view of the platform implemented in TEAMS.

The component, signal, and testing points are shown in the above figures. The internal connection of the function module is the simplified connection state, and the signal indicates the propagation path of the soft error.

**Fig 4:**
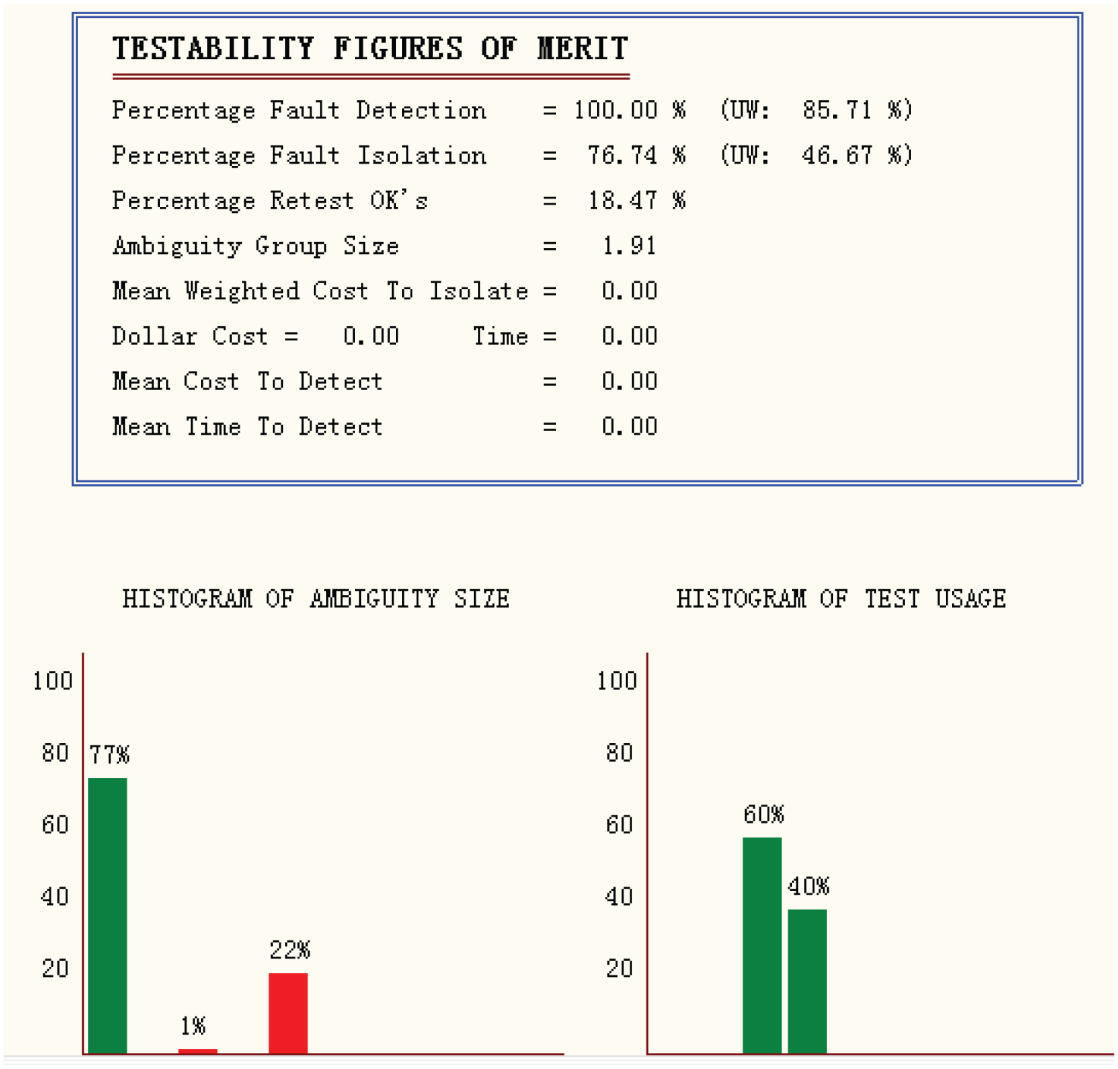
Testing report of the platform.

The testing report of the signal processing baseband is shown in Fig 4. The report includes test options, system statistics, test algorithm statistics, testability figures of merit, histograms of ambiguity sizes, and histograms of test usage. The percentage fault detection is 100%, while the percentage fault isolation is 76.74%.

## Modeling of system functional error rate

The system functional error rate is difficult to define [13]. Three processes can be distinguished from the bombardment of heavy ions to the system functional error. In the first process, transients are generated from the basic physical interaction of single ionizing particles with semiconductor material. This induces the generation of SEE soft errors. The SEE soft errors include single event transient (SET), single event upset (SEU), etc. The second one is the propagation of SEE soft errors. Some of the soft errors may be masked by the structure of the system. For example, Wang et al. showed that 85% of the soft errors in a processor are masked at the architectural level [14]. The third process is the functional error of the system. The functional error means that the actual system behavior is different from the expected behavior. Considering the observability and controllability, we define the output error of the system as its functional error. The model of the system functional error rate is based on the three processes.

One SEU sensitivity parameter which is used to judge the device effect is the SEU static cross section, which was introduced by Elder [15]. The definition is:

$$\begin{array}{c} \sigma =\frac{\text{Number}\_\text{of}\_\text{SEU}\_\text{observed}}{\text{Particle}\_\text{Fluence}}=\frac{N_{seu}}{F} \end{array}$$ (1)

It is the SEU probability induced by a single particle, and F is the total particle influence.

$$\begin{array}{c} F=f\times t \end{array}$$ (2)

where f is the average flux rate of the particle (particle/*cm*^2^/s), and t is the time.

Owing to the masked effect of the system structure, the SEU error may be masked. This means that not all the SEU can induce the system output error.

### Model 1

The system function error rate is:

$$\begin{array}{c} P=\frac{\text{Number}\_\text{of}\_\text{Output}\_\text{Error}\_\text{observed}}{\text{Particle}\_\text{Fluence}}=\frac{N_{sf}}{F} \end{array}$$ (3)

In our experiments, testing equipment was used to monitor the system. Based on the response data, we determined whether the system function error was occurring. We recorded the particle influence and time when the system error is occurred. Many experiments were conducted for heavy ion, and we obtained the average value as the final value. Accordingly, the formula is:

$$\begin{array}{c} \bar{P}=\frac{\sum_{i=1}^{n}P_{i}}{N} \end{array}$$ (4)

Where N is the number of times the test is conducted. Otherwise, support S of the device area is tested by using ions. Thus, the final value is:

$$\begin{array}{c} \hat{P}=\frac{\sum_{i=1}^{n}P_{i}}{N}=\frac{\sum_{i=1}^{n}\frac{N_{sf}}{f\times t}}{N} \end{array}$$ (5)

In our experiments, when each system function error was observed, we recorded the flux rate of particle f and time t. Additionally, the system functional failure mode was recorded.

For every single experiment, the value is:

$$\begin{array}{c} \hat{P}=\frac{1}{f\times t} \end{array}$$ (6)

For N experiments, the value is:

$$\begin{array}{c} \hat{P}=\frac{\sum_{i=1}^{n}\frac{1}{f\times t}}{N} \end{array}$$ (7)

This means that the system functional error probability is induced by one particle. The unit of the SFER is error/particle/*cm*^2^.

The standard deviation of the system functional error rate is:

$$\begin{array}{c} std={\left(\frac{1}{N-1}\sum_{i=1}^{N}{\left(P_{i}-\hat{P}\right)}^{2}\right)}^{\frac{1}{2}} \end{array}$$ (8)

The system functional error rate means the average functional failure probability value of the system being tested.

### Model 2

For a single FPGA-based system, the renowned model to evaluate the SEE vulnerability is [16]:

$$\begin{array}{c} \sigma_{dynamic}=\sigma_{static}\times \varepsilon \end{array}$$ (9)

where *σ*_*dynamic*_ is used to evaluate the system reliability. It is the failure rate of the system per area per particle. *σ*_*static*_ is used to evaluate the SEE device sensitivity. It is the physical parameter of the device and is determined only by the material and structure of the device itself.*σ*_*dynamic*_ depends on the detailed design of the system function on the FPGA.*ε* is the SEE sensitivity factor, which is decided by the utilization resource of FPGA. Further, we can state the model as the following:

$$\begin{array}{c} \sigma_{dynamic}=\sigma_{static}\times R_{res}\times \zeta \end{array}$$ (10)

Where *R*_*res*_ is the utilization resource of the FPGA, and *ζ* is the corresponding factor, which is determined by the system design implementation after placing and routing of FPGA.

The system functional error rate and the dynamic cross section have the same physical meaning. The different models present different calculating algorithms and testing methods.

Model 1 is based on the data of accelerated radiation tests, especially the amount of heavy ions and failure times. It is a black box testing method, because we can calculate the error rate without the information of electronic system.

Model 2 is a gray box testing method for single FPGA circuit. Error rate can be calculated combing static cross section of accelerated radiation tests and the utilization of FPGA.

Both the above two models can not model the interlink state of VLSIs in complex electronic systems, and can not estimate the error rate at the early design phase of the system. Multi-signal flow graph (MSFG) is a white box testing method. It is the advanced and developed method compare to the above two models. More reliability information can be analyzed at the early design phase, more soft error mitigation method can be adopted. Fault diagnosis is analyzed by MSFG, and the error rate is estimated combing the results of accelerated radiation tests and MSFG.

## Experimental System

**Fig 5:**
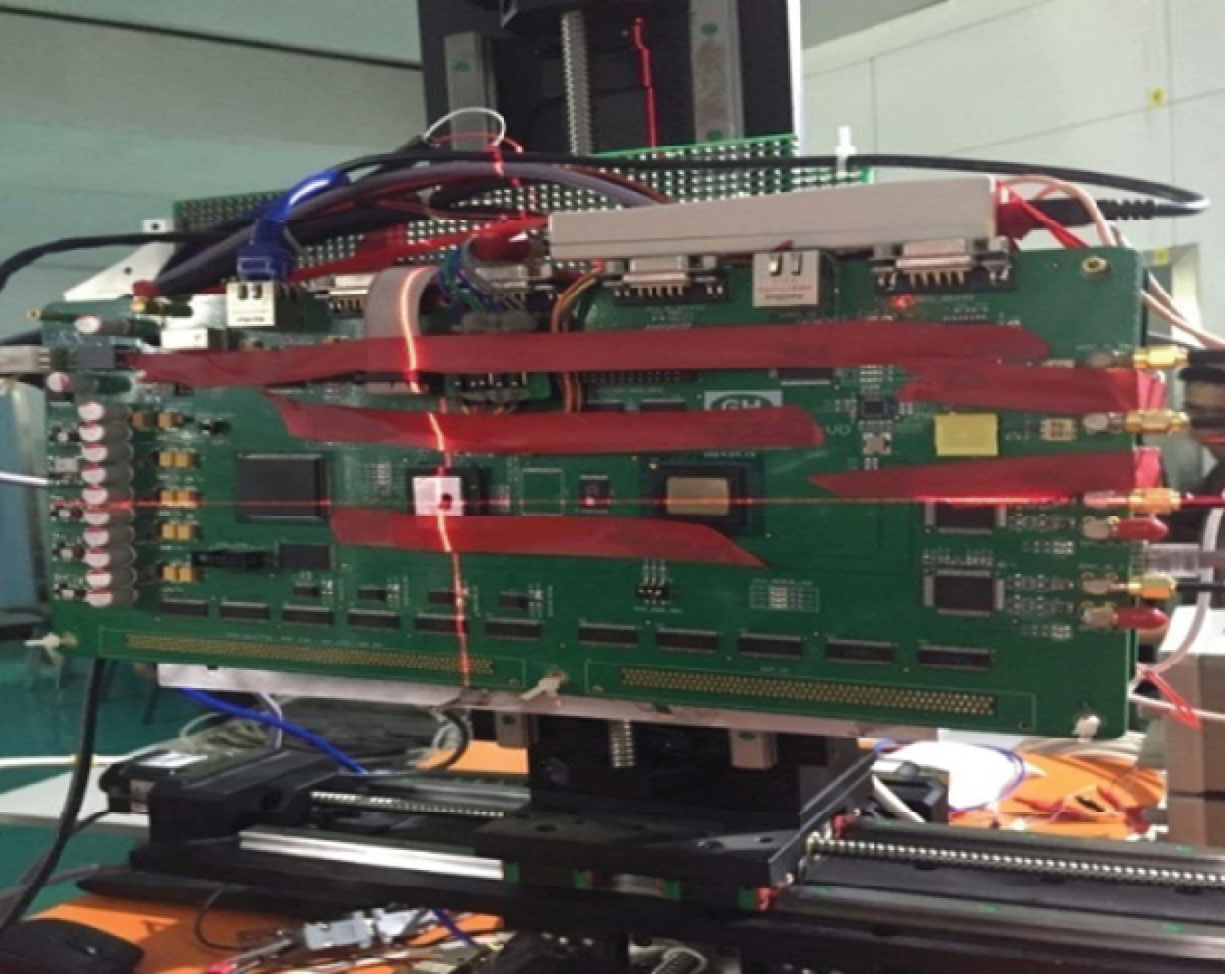
Real testing figure.

**Fig 6:**
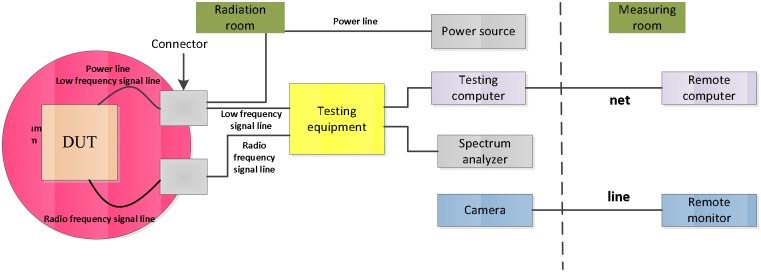
Experimental system.

The experimental system consists of signal processing platform, testing equipment, and heavy ion equipment. The experimental system is shown in Figs 5 and 6.

The signal processing platform is fastened to a special holder in a vacuum room. The device under test (DUT) is a Virtex-II FPGA XC2V1000-BG575, it is the SEE sensitivity device of the signal processing platform. Testing equipment is used to monitor the function of the platform. The corresponding testing line and power line are switched from the vacuum room. They are connected with the testing equipment and power source. One testing computer is used to monitor the experiment.

The above equipment is placed in the radiation room. In the measuring room, a remote computer is connected to the testing computer. The researchers are situated in this room. A remote camera is used to watch the testing scene. The heavy ion equipment is an HI-13 Tandem Accelerator from the Atomic Energy Institute of Physics Science Research Institute in Beijing. The accelerator provides special SEE radiation equipment based on magnetism scanning technology. It provides heavy ions, such as C and Ti, etc., as well as dozens of ions. The XC2V1000 exhibits upsets starting at an LET of approximately 1 MeV.*cm*^2^/mg [17]. Considering the SEE sensitivity of DUT, four ions are chosen. The angle of incidence of the heavy ions is 90-degree peak. Related details are listed in Table 3.

**Table 3:** Main parameter of ions.

Ion	Energy(MeV)	LET(MeV.cm2/mg)	Rang(um)
O	112	2.88	113
Si	156	8.62	60.8
Cl	175	12.6	51.1
Ti	184	21.3	37.7

The Virtex-II-series FPGA is fabricated in a 0.15um/0.12um CMOS eight-layer metal process with a core voltage of 1.5V. The cover of the XC2V1000 is removed before the experiment. The removed area is approximately 32% of the whole wafer. Of the XC2V1000 features, there are 2,787,740 configuration bits, 737,280 block RAM bits, 432 IOBs, 40 multiplier blocks, and eight DCMs. Detailed device information is provided in the Virtex-II platform FPGA handbook.

According to the FPGA function, the device resource utilization is summarized in Table 4.

**Table 4:** FPGA resource utilization summary.

Resources Type	Utilization Amount	Percentage
Number of Slices	3081/5120	60%
Number of Slice Flip Flops	4637/10240	45%
Number of 4 input LUTs	4055/10240	39%
Number of bonded IOBs	130/328	22%
Number of BRAMs	9/40	22%
Number of MULT18X18s	4/40	10%
Number of GCLKs	12/16	75%
Number of DCMs	3/8	37%

## Experimental Results and Discussion

In our experiment, when each system functional error was observed, we recorded the flux rate of particle f and time t. There are 79 group testing data items for four different ions, 24 groups of the O ion, 25 groups of Si, 20 groups of Cl, and ten groups of Ti.

### System functional failure mode

The system functional failure mode is the variously disabled phenomenon of the system. It is an observational feature on the system level. Four main failure modes are classified based on the function of the system, they are uplink remote control error, uplink measuring error, downlink measuring error, and downlink telemetry error. Based on the 79 group testing data items, the statistics of the system functional failure mode are shown in Table 5.

**Table 5:** The system functional failure mode.

Resources Failure Mode	Mount	Probability
Uplink remote control error	29	23.77%
Uplink measuring error	22	18.03%
Downlink measuring error	50	40.98%
Downlink telemetry error	20	16.39%
Other error	1	0.83%
Total	122	100%

Based on the statistics, it is obvious that the downlink measuring error is the main system functional failure mode with a probability of almost 40.98%. The uplink remote control error is 23.77%, which is almost 1/5. The uplink measuring error and downlink telemetry error are both approximately 17%, which is almost 1/6. Other errors can be ignored.

The failure mode mount is larger than that of the testing data because some testing data may induce more than one failure mode. Researchers have shown that a single event upset of configuration memory can induce a multi-block error among key logic designs, possibly even causing the whole key logic design to fail totally. This phenomenon is a single event upset which induces a multi-block error (SEU-MBE) [18]. The upset is induced by the error of programmable route resources. The heavy ions are randomly determined, which means the SEU probability of each bit of FPGA is equal. The different system functional error modes indicate the resource utilization of different functional modules. Hence, the sensitivity of each configuration bit is application-dependent.

Moreover, the working current of the system is observed. The normal value is 0.607A. With the injection of heavy ions, the current value increased; however, no single event latch is observed. This may be on account of the error of inner routing resources of FPGA. The current becomes normal when the system is reset or the FPGA is scrubbed.

### Raw result of different ions

Data of the different ions and flux rates are listed below. The raw results of four ions are shown in Table 6.

**Table 6:** Experimental results of ions.

Ions	Flux ions/s/*cm*^2^	No.	1	2	3	4	5	6	7	8	9	10
O	70	T/s	20	32	8	20	52	20	18	88	43	9
	100	T/s	30	23	27	8	24	21	45	39	14	/
	120	T/s	22	18	35	4	28	/	/	/	/	/
Si	21	T/s	11	31	58	47	89	67	45	63	68	103
	38	T/s	63	32	15	30	29	/	/	/	/	/
	53	T/s	13	21	34	30	21	48	4	23	3	104
Cl	13	T/s	96	188	77	128	152	56	9	27	10	195
	17	T/s	74	77	101	235	39	14	21	61	30	30
Ti	10	T/s	35	170	256	128	26	24	123	9	63	25

### system functional error rate of different ions

According to the testing data presented in Section 4.2 and the formula in Section 3, we can calculate the system functional error rate for different ions. For every single experiment, the system functional error rate is calculated as Formula 6. The average system functional error rate of each flux rate is calculated as Formula 7. For the different flux rates of the ion, the average system functional error rate is also presented. The experimental results are presented in Table 7.

**Table 7:** The experimental result.

Ion	Valid LET	Flux rate	system functional error rate	Average system functional error rate
	(MeV.*cm*^2^/mg)	(ions/s/*cm*^2^)	(error/particle/10^−3^ *cm*^2^)	(error/particle/10^−3^ *cm*^2^)
		70	0.753	
O	2.88	100	0.497	0.644
		120	0.692	
		21	1.192	
Si	8.62	38	0.956	1.341
		53	1.683	
Cl	12.6	13	2.417	
		17	1.579	1.998
Ti	21.3	10	3.014	3.014

**Fig 7:**
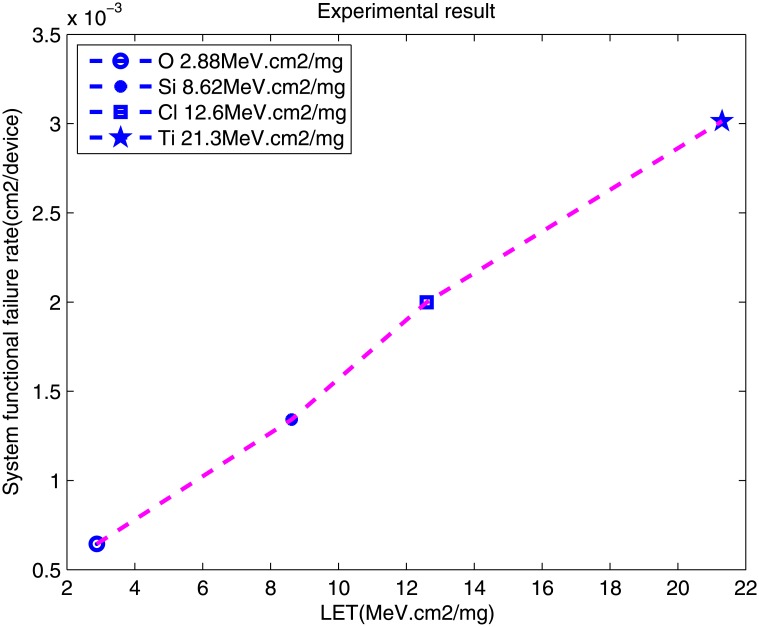
Comparison of SFER and LET.

Furthermore, we can obtain the P-LET curve based on the results. The system functional error rate is approximately 10^−3^(error/particle/*cm*^2^). As shown in Fig 7, with the increase of valid LET, the system functional error rate increases.

#### MTTF estimation based on MSFG

**Fig 8:**
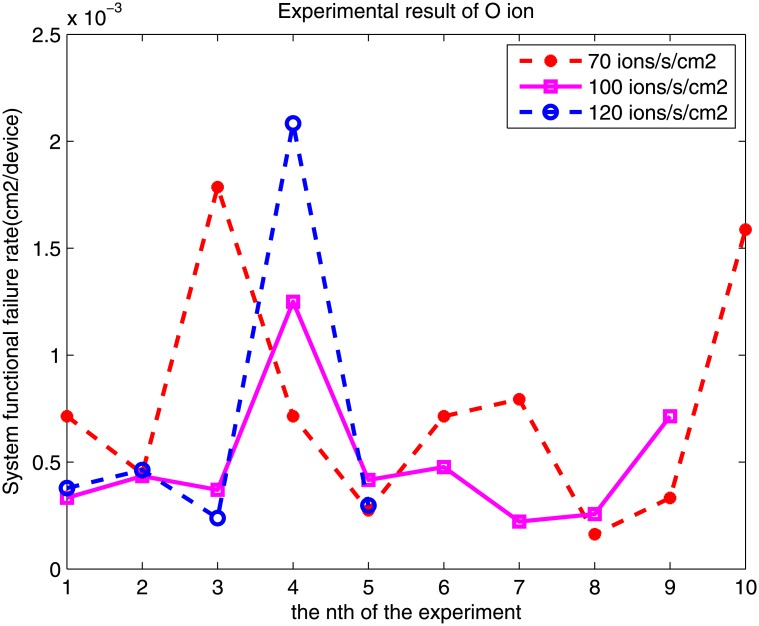
SFER when the O ion is irradiated.

**Fig 9:**
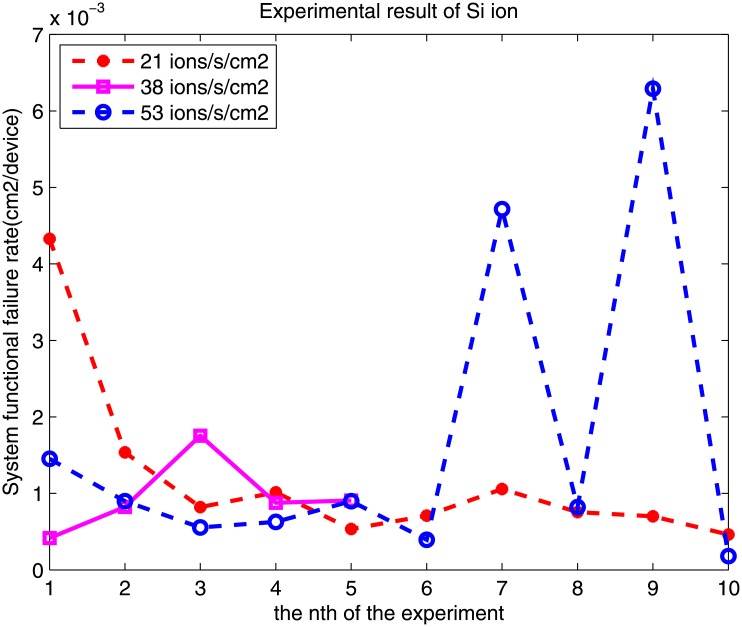
SFER when the Si ion is irradiated.

Based on the MSFG model and the results of accelerated radiation testing, we can evaluate the meantime to the first failure. The results of this experiment indicate that the system functional error rates differ. Taking the experimental results of O and Si ions for examples, the system functional error rate of O ions of each experiment is shown in Fig 8, and the system functional error rate of the Si ion is shown in Fig 9.

For different flux rates of O, the average and standard deviation is

$f=70ions/s/cm^{2},\hat{P}=0.753\times 10^{-3},std=5.393\times 10^{-4}$.
$f=100ions/s/cm^{2},\hat{P}=0.497\times 10^{-3},std=3.165\times 10^{-4}$.
$f=120ions/s/cm^{2},\hat{P}=0.692\times 10^{-3},std=7.823\times 10^{-4}$.

For different flux rate of Si, the average and standard deviation is:

$f=21ions/s/cm^{2},\hat{P}=1.192\times 10^{-3},std=1.1\times 10^{-3}$.
$f=38ions/s/cm^{2},\hat{P}=0.956\times 10^{-3},std=4.883\times 10^{-4}$.
$f=53ions/s/cm^{2},\hat{P}=1.683\times 10^{-3},std=2.1\times 10^{-3}$.

According to Formulas 7 and 9, the system functional error rate should be the same value for a certain FPGA-based system and a certain heavy ion, regardless of the different particles. However, the value is within a certain bound. As shown in Figs 8 and 9, the value occasionally fluctuates. For example, the value of the O ion is approximately 0.001–0.007. For different flux rates, the results are different. The relation between the system functional error rate and the ion flux is neither a positive nor a negative correlation.

There are two factors that may cause the error. One factor is f and the other is t, where f is the average flux of the particle and t is the time. The average flux rate of the particle is provided by the monitor system of the HI-13 Tandem Accelerator. The low flux rate (10–100 ions/s/*cm*^2^) may not be very accurate.

Based on Formula 7, one possible way to increase the accuracy of the result is to ensure the accuracy of the average flux rate of the particle and record time t. Accordingly, we can first record N errors, and then record f and t.

Based on Formula 9, we can evaluate the SEE sensitivity of the FPGA-based system. According to the Virtex-II static SEU characterization [17], the cross section of the XC2V1000 is approximately 10^−8^ per bit/*cm*^2^. Based on the resource utilization of the FPGA, the configuration bits are approximately 106. Wang has shown that 85% of the soft errors in a system are masked at the architectural level. Therefore, the system functional error rate is approximately 10^−3^(10^−8^ × 10^6^ × 85%). This is approximately the same number as in the experimental results. Thus, the experimental results are relatively valid. It indicates that the FPGA is vulnerability to heavy ions, especially after the core cover is removed, on account of the signal processing platform structure and resources utilized by FPGA.

The SEE soft error rate of the component varies with the equivalent linear energy transform (LET) of the particles in the radiation environment. There are three main types of radiation sources: trapped electrons and protons (Van Allen radiation belts), solar protons and ions, and cosmic ray ions. The statistical SEE data indicate that the radiation environment in regular solar conditions is 1000 times of the worst data being at 5min [19].

Based on the MSFG model and the results of accelerated radiation testing shown in Table 7, we can evaluate the meantime to the first failure. Suppose that SEE soft error rate of the HRU is 10^−5^*bit*^−1^ × *day*^−1^. The SRAM-based FPGA and DSP is 10^−3^*bit*^−1^ × *day*^−1^(almost 10^3^ upset *device*^−1^ × *day*^−1^). Based on the multi-signal flow graph model and simulation in TEAMS, the meantime to the first failure is 110.7 h, which is approximately 4.6125days. The results indicate that the FPGA+DSP platform is vulnerable to harsh environments without mitigation. Therefore, it is urgent to adopt mitigation technologies, such as TMR and scrubbing for configuration.

## Conclusion

To evaluate SEE vulnerability of space instrument, MSFG is used to model the complex electronic system, fault-test dependency matrix is modeled, and error rate is evaluated combing the accelerated radiation test. As the knowledge as we know, no literature was found to evaluate the SEE vulnerability of electronic system by this method. It enlarges the knowledge of SEE vulnerability analysis method.

Several approaches have been developed to model the SEE soft error propagation based on MSFG. One approach is that SEE soft error propagation is based on 5 hierarchical levels, they are system level, functional module level, circuit level, device level and fault mode level. It is different with the 9 hierarchical levels of MSFG model. Second approach is that fuzzy probability is proposed to model the soft error propagation probability between modules. In traditional MSFG model, the quantization relationship between two modules is 1 or 0, because it supposes that fault is certain and one fault can lead to another fault. But SEE soft error in complex electronic system is uncertain, fault-coupling. So we introduce fuzzy probability instead of the certain 1 or 0. Third approach is that forward analysis in developed instead of backward analysis. In traditional MSFG model, fault analysis is based on backward analysis. System failure is confirmed at the beginning, and the error source is located by adding test points from top level to down level. However, forward analysis in adopted in SEE soft error propagation model. All the above approaches can overcome the barrier adopt MSFG to model SEE soft error propagation.

For the signal processing platform, we provided a hierarchical partition for the system, classified the fault mode for each component, inserted signals to the components, and added test points. The multi-signal flow graph model was established and simulated in TEAMS software. The percentage fault detection is 100%, and the percentage fault isolation is 76.74%.

The system functional error rate of the signal processing platform was modeled and tested. The XC2V1000 was employed as the SEE sensitivity device of the system. It was affected by the ion with low LET from(2.88 MeV.cm2/mg) to (21.3MeV. *cm*^2^/mg). Based on the experimental results of O, Si, Cl, and Ti ions, the system functional error rate was approximately 10^−3^ (error/particle/*cm*^−2^). Based on the system failure mode, it is obvious that the downlink measuring error is the main system functional failure mode with a probability of almost 40.98%. Based on the MSFG model and the results of accelerated radiation testing, the meantime to the first failure is 110.7 h, which is approximately 4.6125days.

Three main conclusions can be drawn from the results. First, the system functional error rate is primarily determined by the system itself, especially the system implementation details. For the FPGA-based system, the rate is determined by the SEE characteristic of the FPGA, as well as by the system placement and routing. However, more details and an accurate analysis method should be employed to study the problem. Second, different heavy ions correspond to the different system functional error rates. With an increase of the ion LET, the error rate likewise increases. The ion LET of this paper is low; therefore, the high LET of some ions deserves further study. Third, for a certain FPGA-based system, the ion flux is not a factor that affects the result. The experimental results aligns with this theory. Thus, the model of system functional failure rate is valid.

More research can be conducted to enrich the knowledge of the problem discussed herein. First, the ion LET of this paper is low, ranging from (2.88 MeV.*cm*^2^/mg) to (21.3MeV.*cm*^2^/mg), and the ion flux is low, ranging from (10 ions/s/*cm*^2^) to (120 ions/s/*cm*^2^). Ions with high LET and flux rate should be adopted to experiment. Second, more SEE information can be detected during the experiment. Based on the characteristic feature of Xilinx, the information of an SEU of the configuration memory should be detected. For example, the mount and location of the SEU can be detected. Third, a method without radiation ground testing should be proposed to analyze the problem. An analytical method based on the mathematical model of the electronic system can be used to evaluate the SFER.
